# Fructose 2,6-Bisphosphate in Cancer Cell Metabolism

**DOI:** 10.3389/fonc.2018.00331

**Published:** 2018-09-04

**Authors:** Ramon Bartrons, Helga Simon-Molas, Ana Rodríguez-García, Esther Castaño, Àurea Navarro-Sabaté, Anna Manzano, Ubaldo E. Martinez-Outschoorn

**Affiliations:** ^1^Unitat de Bioquímica, Departament de Ciències Fisiològiques, Universitat de Barcelona, Institut d'Investigació Biomèdica de Bellvitge (IDIBELL), Catalunya, Spain; ^2^Centres Científics i Tecnològics, Universitat de Barcelona, Catalunya, Spain; ^3^Department of Medical Oncology, Thomas Jefferson University, Philadelphia, PA, United States

**Keywords:** fructose 2, 6-bisphosphate, cancer metabolism, glycolysis, PFKFB isoenzymes, TIGAR, tumor microenvironment

## Abstract

For a long time, pioneers in the field of cancer cell metabolism, such as Otto Warburg, have focused on the idea that tumor cells maintain high glycolytic rates even with adequate oxygen supply, in what is known as aerobic glycolysis or the Warburg effect. Recent studies have reported a more complex situation, where the tumor ecosystem plays a more critical role in cancer progression. Cancer cells display extraordinary plasticity in adapting to changes in their tumor microenvironment, developing strategies to survive and proliferate. The proliferation of cancer cells needs a high rate of energy and metabolic substrates for biosynthesis of biomolecules. These requirements are met by the metabolic reprogramming of cancer cells and others present in the tumor microenvironment, which is essential for tumor survival and spread. Metabolic reprogramming involves a complex interplay between oncogenes, tumor suppressors, growth factors and local factors in the tumor microenvironment. These factors can induce overexpression and increased activity of glycolytic isoenzymes and proteins in stromal and cancer cells which are different from those expressed in normal cells. The fructose-6-phosphate/fructose-1,6-bisphosphate cycle, catalyzed by 6-phosphofructo-1-kinase/fructose 1,6-bisphosphatase (PFK1/FBPase1) isoenzymes, plays a key role in controlling glycolytic rates. PFK1/FBpase1 activities are allosterically regulated by fructose-2,6-bisphosphate, the product of the enzymatic activity of the dual kinase/phosphatase family of enzymes: 6-phosphofructo-2-kinase/fructose 2,6-bisphosphatase (PFKFB1-4) and TP53-induced glycolysis and apoptosis regulator (TIGAR), which show increased expression in a significant number of tumor types. In this review, the function of these isoenzymes in the regulation of metabolism, as well as the regulatory factors modulating their expression and activity in the tumor ecosystem are discussed. Targeting these isoenzymes, either directly or by inhibiting their activating factors, could be a promising approach for treating cancers.

## Introduction

Otto Warburg, using the Warburg manometer to measure the oxygen consumption in cells, demonstrated that tumor cells showed rapid and intense glycolysis, in which glucose was oxidized into lactate, despite the presence of abundant oxygen (1). This “Warburg effect” is characteristic of proliferating and transformed cells. Warburg postulated that cancer was a result of defects in mitochondrial respiration, which forced the cell to adopt an anaerobic form of energy generation, glycolysis (2). There are different molecular mechanisms that can explain the Warburg effect, including: mitochondrial malfunction (3), oncogenic alterations (4–7), as well as responses to adapt to the tumor microenvironment (TME) (8–10). Even though not all cancer cells have high glycolytic flux (11), the Warburg effect has been observed in most tumor cells and represents an adaptation to support biomass production (10).

At the same time as Otto Warburg, Herbert G. Crabtree studied the heterogeneity of glycolysis in tumors, describing that the magnitude and relationships between respiratory and glycolytic processes were a common feature of uncontrolled proliferation and not specific to malignant tissues (12). He observed considerable variability between respiratory and glycolytic metabolism among different tumors (13). Moreover, he found that glycolytic activity significantly affected the respiration capacity of tumor tissues being respiration and oxidative phosphorylation inhibited by glucose (13). This observation, referred as the “Crabtree effect,” might be advantageous to cancer cells, allowing them to adjust their metabolism to heterogeneous microenvironments in malignant cells, through the glucose-dependent accumulation of essential metabolites, such as serine, phosphoribosyl-pyrophosphate, and glycerol-3-phosphate, which can trigger mitogenic events (11, 14, 15). The Crabtree effect on tumor cells can be eliminated by adding an excess of inorganic phosphate (Pi) (15, 16).

The resurgence of the role of bioenergetics in cancer began in the early 1990s when studies using 2-deoxy-D-glucose (2-DG) in positron emission tomography (PET) showed that most tumors displayed increased glucose uptake in about an order of magnitude higher than that of normal tissue (8). The increased glucose uptake largely depends on the rate of glucose phosphorylation by hexokinases and the upregulation of glucose transporters Glut1 and Glut3 and less often Glut4 (17). More than 90% of primary and metastatic tumors have high glucose uptake, which directly correlates with tumor aggressiveness (8, 18).

For a long time, studies on cancer cell metabolism had focused on investigating a single cell type. However, recent studies have reported a more complex situation in which metabolic heterogeneity within tumors plays a critical role in cancer progression (19–21). Cancer cells display extraordinary plasticity in adapting to changes in their TME, developing strategies to survive and proliferate. Interactions between tumor cells and non-malignant cells of the TME influence cancer initiation and progression as well as patient prognosis (22–24). Local differences in the TME, including acidity and hypoxia, affect cancer cells progression. If located close to blood vessels, cancer cells can proliferate at a higher rate because of the abundant supply of nutrients, growth factors and oxygen. By contrast, if the supply of nutrients and oxygen is reduced, cancer cells rely more on glycolysis, forcing them to develop strategies for survival and proliferation. Thus, it is not surprising that these cells in metabolically deprived environments are usually chemoresistant and have higher malignancy grades (25). Stromal cells, especially cancer-associated fibroblasts (CAFs), influence the homeostasis of the TME. The interactions between TME and cancer cells strongly affect tumor metabolism and growth (26–29). A “two-compartment” model, referred to as the “reversed Warburg effect,” has been proposed as a new perspective of tumor metabolism in which tumor stroma and adjacent host tissues are catabolic, while cancer cells are anabolic (19–21, 28–30). Energy is transferred from the catabolic to the anabolic compartment via the sharing of nutrients, which promotes tumor growth. Although lactate is generally considered a waste product, it has been demonstrated to fuel oxidative metabolism in cancer cells, favoring a symbiosis between glycolytic and oxidative tumor cells (19–21, 28–30). Metabolic reprogramming of cancer and stromal cells involves a complex interplay between oncogenes, tumor suppressors, growth factors and local factors from the TME. These factors can induce the overexpression and increased activity of isoenzymes and other proteins in cancer cells that are different from those found in non-malignant cells (30).

## Reprogramming the glycolytic phenotype of cancer cells

Most tumor cells have a markedly modified energy metabolism in comparison to differentiated cells. Their metabolism, previously based on respiration, changes to another eminently glycolytic, recognized as the glycolytic phenotype (7, 31–34). Several glycolytic genes are usually overexpressed in many tumors and give place to this phenotype (35). This occurs because tumor cells reprogram cellular metabolism increasing the transcription and/or alternative splicing of glycolytic genes induced by the Hypoxia Inducible Factor-1α (HIF-1α), oncogenes and inactivated tumor suppressor genes and distinct growth factors (34, 36–39). The glycolytic phenotype is a distinctive characteristic of tumor cells (7, 31, 33), providing advantages to proliferating cells and allowing them to metabolize the most plentiful nutrient, glucose, to generate energy and anabolic precursors. Even though the yield of ATP per glucose molecule consumed is low, the percentage of cellular ATP generated from glycolysis can surpass that produced from oxidative phosphorylation if the glycolytic flux is high enough (11, 32, 40). Furthermore, glucose metabolism provides intermediates that are needed for biosynthetic pathways, such as ribose sugars for nucleotide synthesis and hexose sugar derivatives, glycerol and citrate for lipid production, non-essential amino acids (serine, glycine, and cysteine) and NADPH. Therefore, the Warburg effect has a positive impact on bioenergetics, biosynthesis and detoxification of reactive oxygen species (ROS) (10).

**Figure 1:**
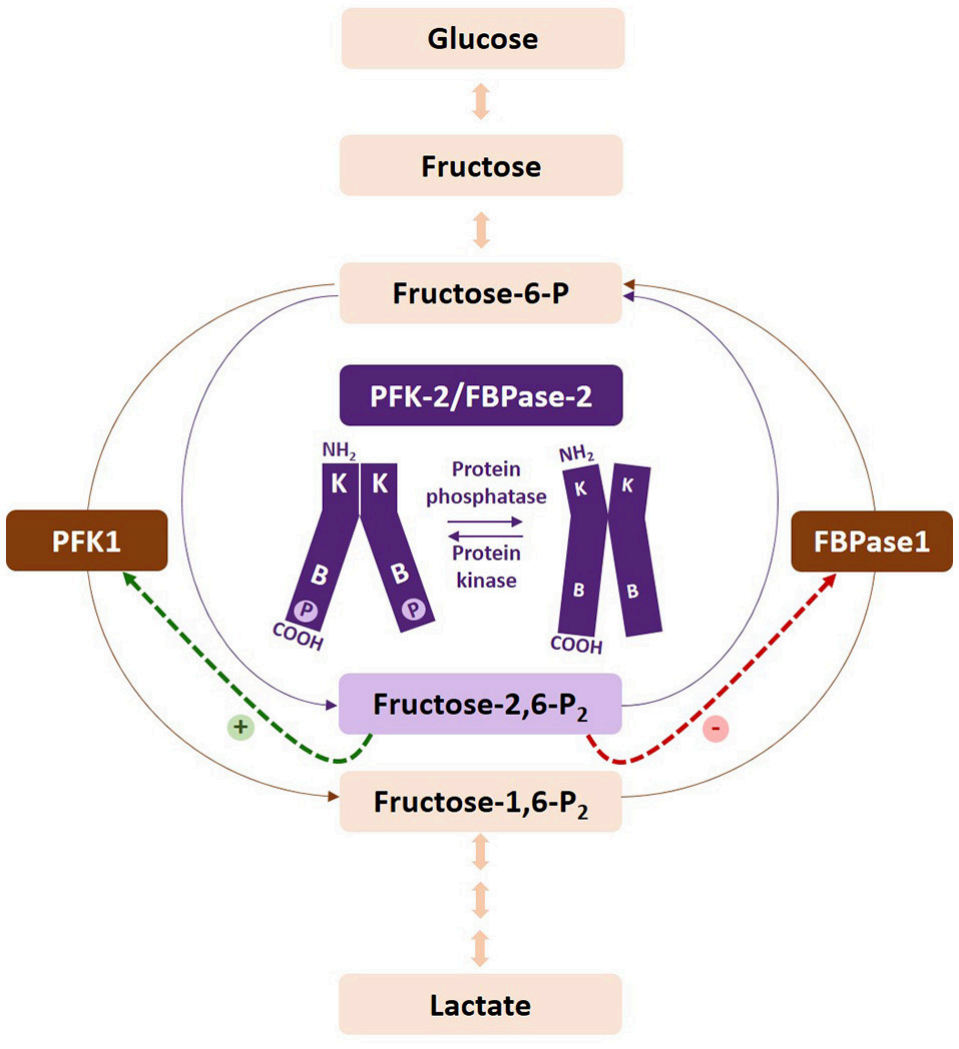
Regulation of the fructose-6-P/fructose-1,6-P_2_ substrate cycle. Fructose-2,6-P_2_ is synthesized and degraded by PFK-2/FBPase-2, respectively. Increased levels of the metabolite activate PFK-1 and inhibit FBPase1, increasing the glycolytic flux. Phosphorylation of the PFKFB2 and PFKFB3 isoenzymes increases their kinase activity. The kinases and phosphatases responsible for their regulation vary according to the isoenzyme. PFKFB1 is not represented by this figure, as phosphorylation increases the bisphosphatase activity of the enzyme. At present, PFKFB4 has not been found to be regulated by phosphorylation.

There are several checkpoints regulating the acquisition of the glycolytic phenotype. The first point of commitment of glucose-6-phosphate to the glycolytic pathway involves the fructose-6-phosphate/fructose-1,6-bisphosphate cycle (Fru-6-P/Fru-1,6-P_2_) (41) (Figure 1). The following paragraphs describe the specific isoenzymes regulating this substrate cycle, the main properties of which are summarized in Table 1.

**Table 1:** Properties of the glycolytic enzymes acting on Fru-6-P/Fru-1,6-P_2_ substrate cycle.

**Protein**	**Gene**	**Chromosomal location (human)**	**Kinase/phosphatase ratio**	**Isoenzymes**	**Regulation**
6-phosphofructokinase (PFK1)	*PFK-M*	12q13.11	Only kinase	PFK-M (muscle)	PKA, PKC and AMPK in rabbit, rat, and pig, respectively
	*PFK-L*	21q22.3	Only kinase	PFK-L (liver)	Ras, Src, HIF1α
	*PFK-P*	10p15.2	Only kinase	PFK-P (platelets)	Akt, EGFR, HIF-1α Glycosylation, Snail
Fructose-1,6-bisphosphatase (FBPase)	*FBPase1*	9q22.32*	Only phosphatase	FBPase1	Snail
	*FBPase2*	9q22.32*	Only phosphatase	FBPase2	Not described
6-phosphofructo-2-kinase/fructose-2,6-bisphosphatase (PFK-2/FBPase-2)	*PFKFB1*	Xp11.21	2.5 (rat liver) 1.2 (bovine liver) 0.4 (rat muscle)	lPFK-2 (liver) mPFK-2 (muscle) fPFK-2 (fetal)	PKA PP-1
	*PFKFB2*	1q32.1	1.8 (bovine heart)	hPFK-2 (heart)	AMPK, PKA, Akt, PKC, RSK Glucocorticoids, androgens, RNA LINC00092
	*PFKFB3*	10p15.1	710 (human placenta) 3.1 (bovine brain)	uPFK-2 (ubiquitous) iPFK-2 (inducible)	AMPK, PKA, Akt, PKC, Smad, RSK, p38-MK2 Estrogens, adenosine, LPS p53, S-glutathionylation, demethylation
	*PFKFB4*	3p21.31	4.1 (rat testis) 0.9 (human testis)	tPFK-2 (testis)	HIF-1α, P-PPARγ, testosterone
					p53
TIGAR	*C12orf5*	12p13.32	Only phosphatase	Not described	p53, CREB, HIF-1α, SP1

Information regarding gene name, chromosomal location, isoenzymes, enzymatic activity, and regulation of the glycolytic enzymes acting on Fru-6-P/Fru-1,6-P2 substrate cycle is shown. Positive and negative regulators are written in green and red, respectively. *Same chromosomal region, different locus.

### 6-phosphofructo-1-kinase (PFK1)

PFK1 is a tetrameric protein, with three genes encoding the PFK-M (muscle), PFK-L (liver), and PFK-P (platelet) human isoforms, with a molecular weight of their subunits of 82.5, 77, and 86.5 kDa, respectively (42). The different isoforms can form homo- or hetero-tetramers depending on the cell type (38, 42). Lactate induces the dissociation of the tetramers into dimers, reducing enzymatic activity and providing negative feedback in the regulation of the glycolytic rate (43). PFK1 isozymes can be phosphorylated by different kinases but this does not produce significant catalytic effects (41, 44, 45), although the covalent modification can affect their association with other proteins (46–48). Localization to the actin filaments of the cytoskeleton has been shown to increase PFK-M activity (49), which can also bind to and modulate protein phosphatase-1 (50). PFK-L, but not PFK-M and PFK-P, assembles into filaments, with fructose-6-P binding essential for their formation (51). These filaments have been observed to localize in the plasma membrane, where they promote the vessel sprouting of endothelial cells (52). PFK-L forms clusters in human cancer cells and colocalizes with other rate-limiting enzymes in both glycolysis and gluconeogenesis, supporting the formation of multienzyme metabolic complexes for glucose metabolism, integrating PFK-L, FBPase1, the pyruvate kinase isoenzyme M2 (PKM2) and phosphoenolpyruvate carboxykinase 1 (PEPCK1), among others, and forming the “glycosome” (53). PFK1 is also regulated by different allosteric effectors, which provides control of the glycolytic flux and coordination of glucose entry into glycolysis. It is a tightly-regulated enzyme and its kinetic and regulatory characteristics depend on the composition of its different subunits. Its regulation involves a series of negative (citrate, ATP, phosphoenolpyruvate, and [H^+^]) and positive effectors (Fru-2,6-P_2_, AMP, Fru-1,6-P_2_, Glu-1,6-P_2_, NH4^+^, and Pi) (41, 42), which coordinate its response to the energy status of the cell. PFK1 activity increases in response to proliferation signals alongside elevated glycolysis in proliferating and cancer cells (54), although there are exceptions where its activity does not increase (44, 54). The main isoenzymes expressed in tumor cells are PFK-P and PFK-L (54, 55). In human lymphomas and gliomas, PFK1 is less sensitive to inhibition by ATP and citrate, and more sensitive to activation by Fru-2,6-P_2_ and AMP (44, 56). PFK1 activity is induced by HIF-1α (57) or the overexpression of the oncogenes RAS and SRC (58). PFK-L and PFK-P can be glycosylated in response to hypoxia, which inhibits PFK1 activity and redirects the glucose flux toward the pentose phosphate pathway (PPP), providing pentose sugars for nucleotide synthesis and NADPH to combat oxidative stress (59). Similarly, the transcription repressor Snail reprograms glucose metabolism by repressing PFK-P, suppressing lactate production and amino acids biosynthesis, while promoting cancer cell survival under metabolic stress (60). Akt can bind to and phosphorylate PFK-P at S386, which inhibits the binding of TRIM21 E3 ligase to PFK-P and its subsequent polyubiquitylation and degradation. This has been shown to increase PFK-P activity, glycolysis, cell proliferation and brain tumor growth (48). Recently, EGFR activation has been reported to elicit lysine acetyl-transferase-5-mediated PFK-P acetylation and subsequent translocation of PFK-P to the plasma membrane, where EGFR phosphorylates PFK-P at Y64. Phosphorylated PFK-P binds to the N-terminal domain of p85α and promotes the activation of PI3K and Akt, leading to increased PFKFB2 activity, Fru-2,6-P_2_ synthesis and PFK1 activation, which in turn promote cell proliferation and tumorigenesis (61).

### Fructose 1,6-bisphosphatase (FBPase1)

FBPase1, a rate-limiting enzyme that catalyzes the opposite reaction to that of PFK1 in the Fru-6-P/Fru-1,6-P_2_ cycle, exists as two isoenzymes in mammals: FBPase1 and FBPase2. Both isozymes are inhibited by Fru-2,6-P_2_ synergistically with AMP (41, 62, 63) (Figure 1). FBPase1 can be phosphorylated by different kinases, but this leads to non-significant catalytic effects (41). FBPase1 is ubiquitously expressed and has been reported to be lost in several human cancers (64). FBPase1 overexpression suppresses cancer cell growth (65) and its loss correlates with advanced tumor stage and poor prognosis (66). Snail can repress FBPase1 in breast cancer cells (67), thus tightly controlling glucose flux through the PPP, by suppressing both PFK-P and FBPase1. FBPase1 and PFK-L directly interact forming multienzyme complexes that can modulate their activities (53). By contrast, FBPase2 is restricted to muscle cells and participates in the synthesis of glycogen from carbohydrate precursors (62).

### 6-phosphofructo-2-kinase/fructose 2,6-bisphosphatase (PFK-2/FBPase-2) isoenzymes

**Figure 2:**
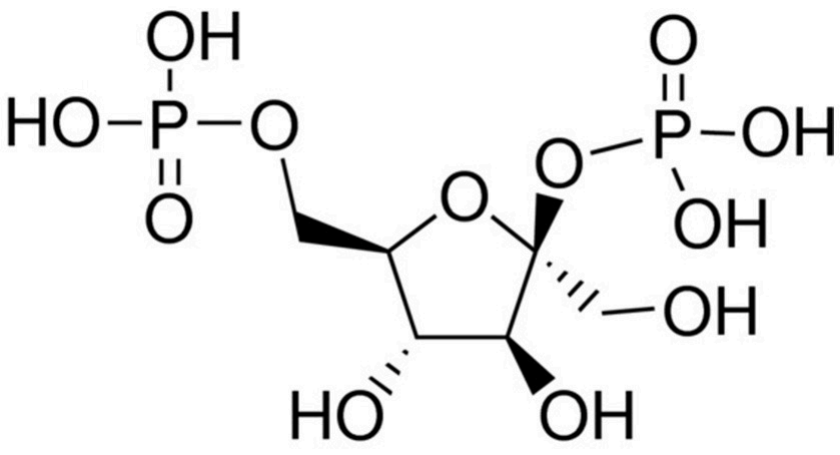
Structure of β-Fructose 2,6-bisphosphate.

Fru-2,6-P_2_ (Figure 2), a powerful allosteric modulator of the Fru-6-P/Fru-1,6-P_2_ substrate cycle, was discovered in 1980 when its concentration was observed greatly increased in hepatocytes upon incubation with glucose and disappeared in the presence of glucagon, providing a refined regulatory mechanism between glycolysis and gluconeogenesis (41, 68–71) (Figure 2). Fru-2,6-P_2_ has a dual function, increasing the affinity of PFK1 for Fru-6-P and releasing the enzyme from ATP-mediated inhibition. It also synergistically increases the affinity of PFK1 for AMP, a positive allosteric effector of the enzyme. By contrast, both Fru-2,6-P_2_ and AMP inhibit FBPase1 (41, 68–71). Furthermore, Fru-2,6-P_2_ stabilizes PFK1 (68) and promotes its association into tetramers and higher oligomers with enhanced activity (72). Therefore, changes in the concentration of this metabolite regulate the activities of PFK1 and FBPase1, thereby conferring a key role to Fru-2,6-P_2_ in the regulation of the Fru-6-P/Fru-1,6-P_2_ substrate cycle and the intensity and direction of glycolysis and gluconeogenesis (Figure 1). Since Fru-2,6-P_2_ does not take part as an intermediary in any metabolic interconversion, and given its lability in acid extracts used to measure phosphoric acid esters in tissues, this metabolite managed to escape discovery until 1980 (41, 70). Fru-2,6-P_2_ has been shown to carry out a leading function in regulating glycolysis in other eukaryotic cells (41, 68, 69).

**Figure 3:**
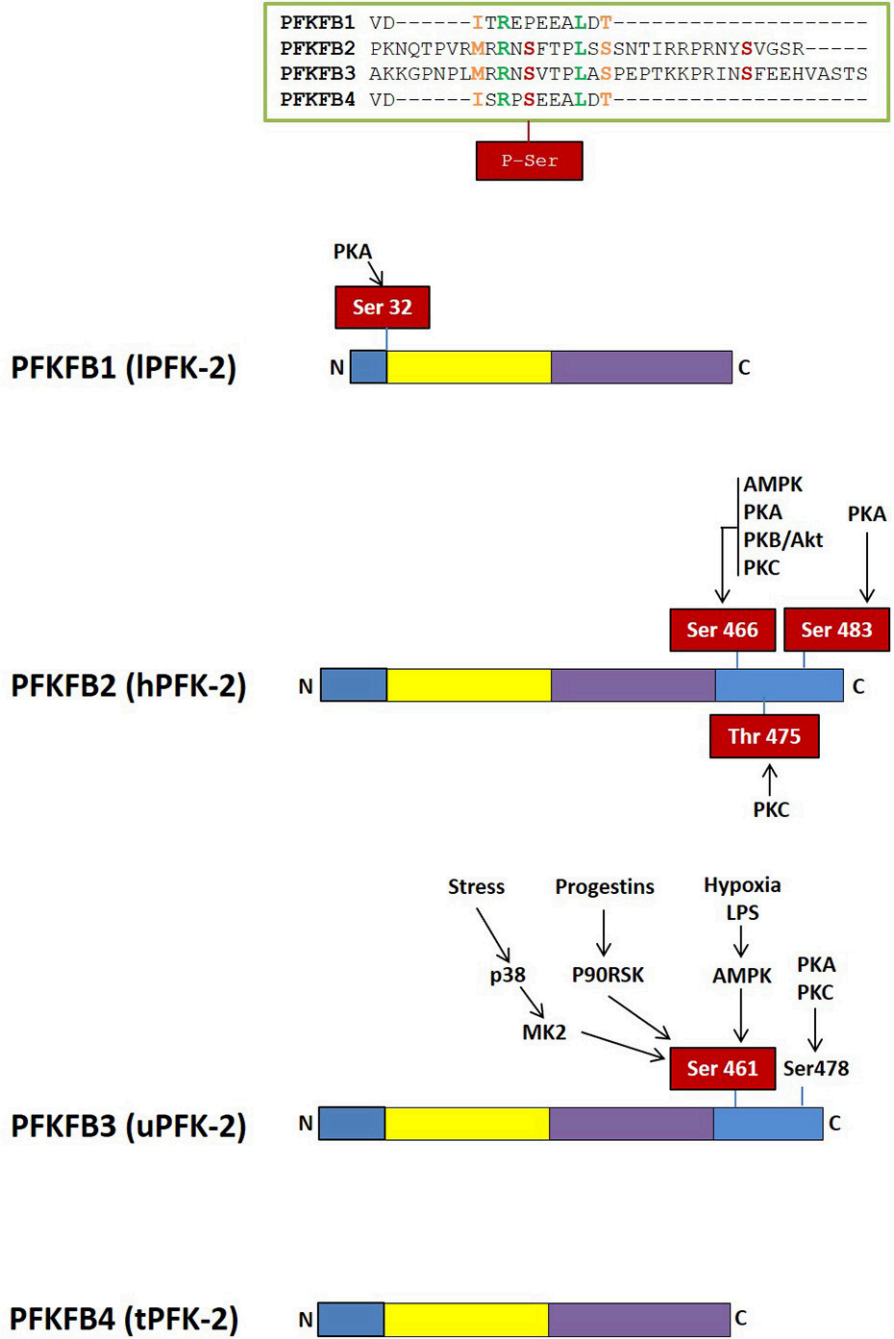
Domain organization of PFK-2/FBPase-2 isoenzymes. The N-terminal PFK-2 domain is shown in yellow and the C-terminal FBPase-2 domain is in purple. Regulatory regions with residues susceptible of phosphorylation by different protein kinases are shown in blue.

Fru-2,6-P_2_ concentration is significantly higher in tumor cells than in normal cells (56, 73, 74) and is regulated by different bifunctional isoenzymes called 6-phosphofructo-2-kinases/fructose 2,6-bisphosphatases (PFK-2/FBPase-2), which catalyze the synthesis and degradation of this metabolite (68–71, 75). The balance between the activity of 6-phosphofructo-2-kinase (PFK-2), which synthesizes Fru-2,6-P_2_ from Fru-6-P and ATP, and that of fructose 2,6-bisphosphatase (FBPase-2), which hydrolyzes Fru-2,6-P_2_ into Fru-6-P and inorganic phosphate, ultimately determines the concentration of this metabolite (Figure 1). PFK-2/FBPase-2 is one of the few homodimeric bifunctional enzymes, composed of two 55-kDa subunits. Each monomer presents both kinase and bisphosphatase domains in the same polypeptide chain, with the kinase domain at the N-terminal end of the protein and the bisphosphatase domain at the C-terminal (68–71, 75, 76) (Figure 3). The amino acids located near the N- and C-terminal ends of the PFK-2/FBPase2 isoenzymes protein are responsible for its post-translational regulation as they can be phosphorylated by different protein kinases. The protein is derived from the fusion of two genes that express a kinase domain, evolutionarily related to the family of proteins that link mononucleotides, and a bisphosphatase domain, which is related to the family of phosphoglycerate phosphatases and acid phosphatases (75–77). The regulatory function of Fru-2,6-P_2_ in carbohydrate metabolism implies that the modulation of Fru-2,6-P_2_ synthesis and degradation must be very well compensated to adapt Fru-2,6-P_2_ concentration to the needs of the cell. The degree of complexity involved in regulating Fru-2,6-P_2_ levels in each tissue and physiological condition is reflected by the existence of different PFK-2/FBPase-2 isoenzymes that are capable of adapting to different conditions (75, 76, 78). These isoenzymes, encoded by four genes (*PFKFB1-PFKFB4*), display variances in their kinetic properties and distribution, as well as in their responses to allosteric, hormonal, and growth factors (75, 76). The *PFKFB1* gene encodes the isoforms that were originally identified in the liver, muscle and fetal tissue, while the *PFKFB2* gene encodes the isoenzyme occurring in the heart and kidney and in some cancer cells. The *PFKFB3* gene encodes the isoforms present in the brain, placenta and adipose tissue, and is the most expressed *PFKFB* gene in proliferating and cancer cells. Finally, the *PFKFB4* gene encodes the isoenzyme occurring in the testis, although it has also been found in several types of tumor cells (Table 1).

#### PFKFB1

The *PFKFB1* gene was cloned from rat and human liver (71, 79, 80) and is composed of 60,944 bp. It contains 17 exons under the control of different promoters and gives rise to three different transcripts, mRNAs L (liver), M (muscle), and FL (fetal) (68, 70, 75, 81). The muscle and fetal transcripts have the same sequence as that of the liver, except for the exon encoding the N-terminal end containing the S32 residue, that can be phosphorylated by the cAMP-dependent protein kinase (PKA) in response to glucagon and dephosphorylated by protein phosphatase 2A (PP2A), activating the bisphosphatase and inhibiting the kinase activities of the liver isoenzyme, respectively (68, 70, 82) (Figure 3). The FL mRNA variant, present in several rat-derived cell lines and proliferating tissues, contains two non-coding exons (1aF and 1bF) (83). Although the liver, muscle and fetal isoenzymes come from the same gene, they are regulated differently, since glucagon induces glucose synthesis in the liver but not in other tissues. *PFKFB1* has not been found to be overexpressed in cancer cells.

#### PFKFB2

The human *PFKFB2* gene was cloned from human heart and it contains 15 exons spanning 27,961 bp. This gene generates nine transcripts, four of which encode full-length proteins, two encode truncated proteins and the other three contain an open reading frame without producing any protein (84). PFKFB2 is a homodimeric protein, with isoform A being a 58-kDa protein containing 505 amino acids and isoform B a 54-kDa protein containing 471 amino acids. The sequence of the catalytic site is preserved, but those of the N- and C-terminal regions exhibit more variances (75, 76, 84, 85). *PFKFB2* is mainly expressed in the heart, being also located in other tissues, but in lesser proportion (76, 86). Moreover, it is expressed in cancer cells from different origins (76, 86, 87).

PFKFB2 can undergo multisite phosphorylation, integrating signals from many pathways (Figure 3). The C-terminal domain residues S29, S466, T475 and S483 can be phosphorylated by protein kinases such as 3-phosphoinositide-dependent kinase-1 (PDPK-1), AMP-activated protein kinase (AMPK), PKA, protein kinase B (PKB; also known as Akt), mitogen-activated protein kinase 1 (MAPK-1), and p70 ribosomal S6 kinase (S6K1). PFKFB2 phosphorylation at three conserved residues (S466, T475, and S483) results in the activation of the enzyme, decreasing its Km for Fru-6-P and increasing the Vmax of PFK-2 activity (75). PFK-2 activity, however, varies depending on the kinase that activates it (75, 88). Moreover, it has been proposed that the 14-3-3 proteins, which promote cell survival (89), bind to PFKFB2 when it is phosphorylated at S483 by Akt in response to insulin and IGF-1 or when transfected with active forms of Akt, mediating growth factors-induced glycolysis (90). Oncogenic BRAF V600E has also been found to activate p90 ribosomal S6 kinase (RSK), which phosphorylates and activates PFKFB2, that then binds to 14-3-3 to promote glycolysis and melanoma cell growth (91). Furthermore, amino acids increase Fru-2,6-P_2_ synthesis and glycolysis in cardiomyocytes and cancer cell lines by PI3K and Akt-mediated phosphorylation of PFKFB2 at S483 (92). Moreover, EGFR activation induces PFK-P phosphorylation (Y64), which binds to the N-terminal SH2 domain of p85α and promotes Akt-dependent PFKFB2 phosphorylation (S483), glycolysis, cell proliferation and brain tumorigenesis (61). Adrenalin promotes PFKFB2 phosphorylation by PKA at S466 and S483, while AMPK activation during ischemia or hypoxia induces PFKFB2 phosphorylation at S466, which increases Fru-2,6-P_2_ levels and stimulates glycolysis (75, 88). PFKFB2 is also a substrate of PKC, which phosphorylates S84, S466, and T475 residues (75, 93) (Figure 3). Several studies have reported that HIF-1α can regulate PFKFB2 expression *in vivo* but this appears to be cell-specific (86). Citrate, whose concentration is increased by the oxidation of fatty acids and ketone bodies, competitively blocks Fru-6-P binding, down-regulating *PFKFB2* expression and glycolysis through the “glucose-sparing effect” (85).

*PFKFB2* is one of the genes increased in lymphoblasts from glucocorticoid (GC)-treated children suffering from acute lymphoblastic leukemia (94). Surprisingly, overexpression of the two *PFKFB2* splice variants seems to have little effect on lactate and ATP production, two metabolites that are reduced after GC treatment, and cell survival, suggesting that this gene is not an essential regulator of the anti-leukemic effects of GC (95). The androgen receptor (AR) is a key regulator of prostate growth, promoting glycolysis and anabolic metabolism, and the principal drug target for the treatment of prostate cancer (96). One of the mechanisms behind this phenotype is the transcriptional upregulation of *PFKFB2*, possibly under the control of the AR-CAMKII-AMPK signaling pathway (96). Androgens have been shown to stimulate glycolysis for *de novo* lipid synthesis. Androgens promote transcriptional up-regulation of *de novo*, mediated by binding of ligand-activated AR to its promoter, and phosphorylation of PFKFB2 generated by the PI3K/Akt signaling pathway. Moreover, blocking *PFKFB2* expression with siRNA or inhibiting PFK-2 activity with LY294002 (PI3K inhibitor) has been observed to reduce glucose uptake and lipogenesis, suggesting that the induction of *de novo* lipid synthesis by androgens requires the transcriptional up-regulation of *PFKFB2* (97). *PFKFB2* expression is also enhanced in human gastric malignant tumors, being associated with increased levels of HIF-1α dependent genes, vascular endothelial growth factor (VEGF) and Glut1, indicating that HIF-1α could be responsible for the induction of *PFKFB2* expression (87). In hepatocellular carcinoma, high expression of metastasis-associated in colon cancer protein 1 (MACC1), a key regulator of the hepatocyte growth factor (HGF)/c-Met pathway, has been noted to correlate with the high expression of *PFKFB2*, this correlation being associated to TNM stage (classification of malignant tumors), overall survival and Edmondson-Steier classification (98). Furthermore, MAPK-activated RSK, which directly phosphorylates PFKFB2, is required to maintain glycolytic metabolism in BRAF-mutated melanoma cells. RSK inhibition reduces PFKFB2 activity and glycolytic flux, suggesting an important role for RSK in BRAF-mediated metabolic rewiring (91).

Another observation highlighting the importance of PFKFB2 in metabolic reprogramming is its contribution to osteosarcoma development. Slit guidance ligand 2 (SLIT2) binds to round about guidance receptor 1 (ROBO1) and plays important roles in various physiological and pathological conditions, such as axon guidance, organ development, and angiogenesis (99). The SLIT2/ROBO1 axis promotes proliferation, inhibits apoptosis and contributes to the Warburg effect in osteosarcoma cells via activation of the SRC/ERK/c-MYC/PFKFB2 pathway (99).

*PFKFB2* expression has also been linked to the regulation of non-coding RNAs. In ovarian cancer, the long non-coding RNA LINC00092 has been identified as a nodal driver of CAF-mediated metastasis. The pro-metastatic properties of CAFs have been linked to the elevated expression of both the chemokine *CXCL14* and *PFKFB2*, correlating with poor prognosis. Mechanistic studies have demonstrated that LINC00092 binds PFKFB2, thereby promoting metastasis by inducing a glycolytic phenotype in these tumors and sustaining the local supportive function of CAFs (100). Similarly, the long non-coding RNA UCA1/miR-182 has been observed to be a nodal driver of metastasis in glioma that is mediated by glioblastoma-associated stromal cells (GASCs) and the GASC-secreted chemokine CXCL14. In clinical specimens, CXCL14 upregulation in GASCs cells was seen to correlate with poor prognosis. Interestingly, GASCs expressing high levels of CXCL14 have been shown to upregulate lncRNA UCA1 and downregulate miR-182, with miR-182 directly binding to PFKFB2 to modulate CXCL14 secretion, glycolysis, and the invasion of glioma cells (101).

PFKFB2 has also important roles in the physiology of human CD3+ T cells. Treatment of activated human CD3+ T cells with the proinflammatory chemokine CCL5 induces the activation of AMPK and PFKFB2 phosphorylation and activation, promoting glycolytic flux and suggesting that both glycolysis and AMPK signaling are required for efficient T cell activation in response to CCL5 (102).

#### PFKFB3

The *PFKFB3* gene was cloned from a cDNA library of fetal brain and has been found expressed in all the tissues studied (103–107). It spans 109,770 bp and is composed of 19 exons. The variable C-terminal domain can undergo alternative splicing to produce six different isoforms. The two main isoforms are generated by alternative splicing of exon 15 and differ in their C-terminal sequence, the 4,553 bp mRNA variant initially named as ubiquitous PFK-2 (uPFK-2) (107) and the 4,226 bp mRNA inducible PFK-2 (iPFK-2) variant (106). Four additional splice variants have also been described (108). As many proto-oncogenes and pro-inflammatory cytokines, PFKFB3 has multiple copies of the AUUUA sequence in the 3′UTR of its mRNA, which confer instability and enhanced translational activity (106). It has been found that miR-26b and miR-206 interact with the 3′UTR of the PFKFB3 mRNA, decreasing glycolysis in osteosarcoma and breast cancer cells, respectively (109, 110).

*PFKFB3* gene expression is induced by different stimuli, such as hypoxia (31, 111, 112), progestin (104, 113), estrogens (114) and stress stimuli (115), through the interactions of HIF-1α, the progesterone receptor (PR), estrogen receptor (ER), and the serum response factor (SRF). These factors bind to specific sequences in PFKFB3 promoter which are the consensus hypoxia response element (HRE), the progesterone response element (PRE), estrogen response element (ERE), and the serum response element (SRE), respectively. *PFKFB3* expression can also be stimulated by growth factors such as insulin (73), pro-inflammatory molecules (106) such as interleukin-6 (IL-6) (116, 117), lipopolysaccharide (LPS) and adenosine (118), mitogenic lectins such as concanavalin A (ConA) (119) and phytohemagglutinin (PHA) (120), and the transforming growth factor beta 1 (TGF-β1) (121).

*PFKFB3* gene expresses an isoenzyme that has high kinase and low bisphosphatase activity (K/B = 710), favoring the net synthesis of Fru-2,6-P_2_ and eliciting high concentrations of this metabolite in proliferating and tumor cells (122). The presence of a serine instead of an arginine at position 302 in the bisphosphatase active site gives place to the low bisphosphatase activity (123).

**Figure 4:**
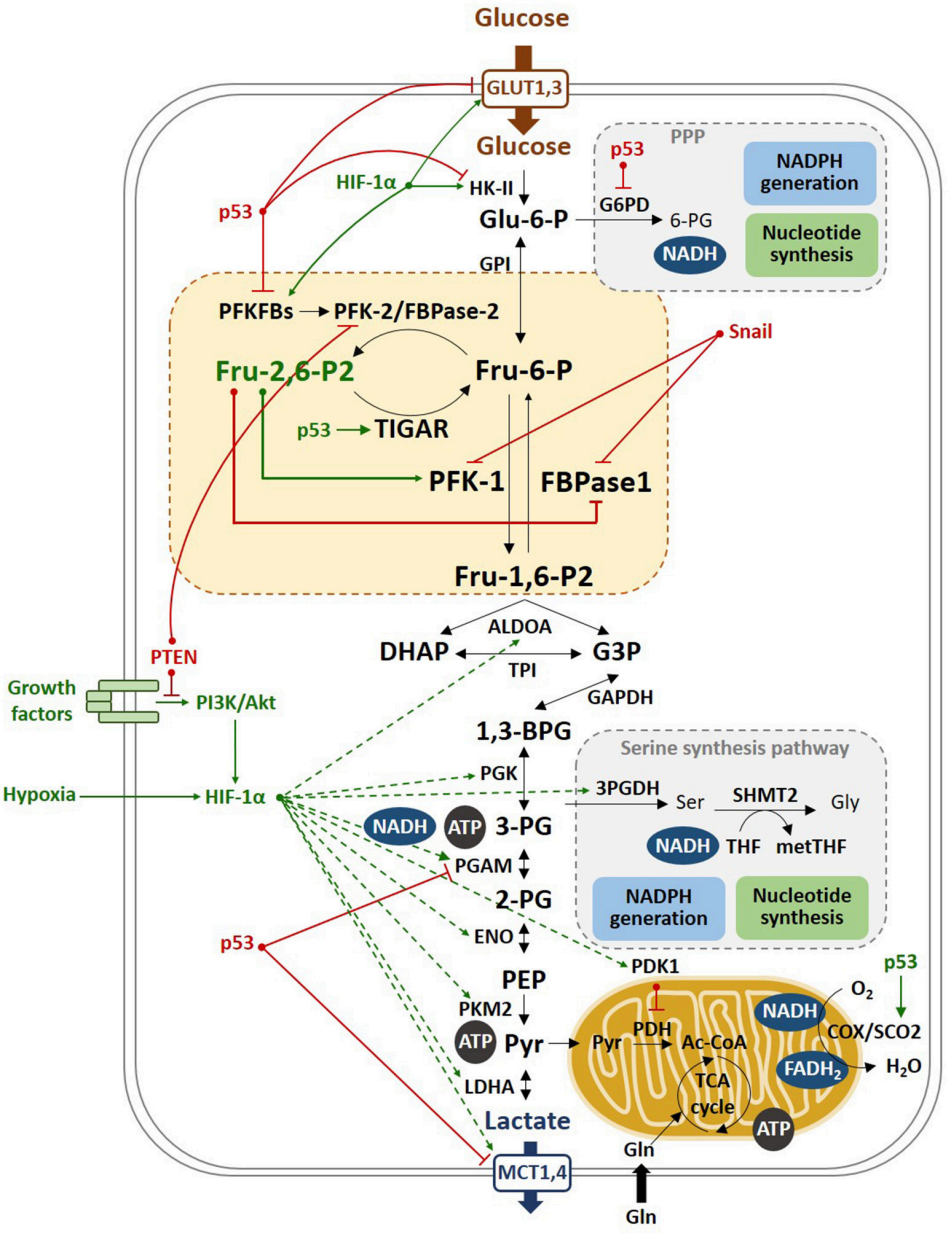
Molecular mechanisms of the Warburg effect. Hypoxia stabilizes HIF-1α, which transactivates most of the glycolytic genes, such as HK-II and PFKFBs, as well as PDK1, which in turn inhibits PDH, that catalyzes the conversion of pyruvate to Ac-CoA. PDH inhibition blocks the entry of pyruvate into the TCA, promoting the oxidation of pyruvate to lactate. Lactate is then excreted through the MCT transporters, which expression is also increased by HIF-1α. p53 inhibits the glycolytic genes PFKFB3, PFKFB4, and PGAM and the lactate transporter MCT1, and induces TIGAR, reducing the glycolytic flux. This results in increased flux of the pentose phosphate shunt to produce NADPH and ribose-5P. Moreover, p53 stimulates respiration by inducing SCO2, a component of cytochrome c oxidase (COX). In tumors, limited access to oxygen and mutations in the p53 gene drive to increased glycolysis, a phenomenon named as the Warburg effect.

Different protein kinases, such as AMPK (124, 125), RSK (113), MK2 (115), PKA, PKB (119), and PKC (125) regulate the PFKFB3 isoenzyme by covalent modification of its C-terminal domain (Figure 3). PI3K/Akt also controls the PFKFB3 isoenzyme downstream of growth factors signaling (119, 126, 127). Phosphorylated PFKFB3 has increased Vmax of its kinase activity and decreased Km for fructose-6-P (119, 124). ROS-mediated S-glutathionylation (128) or demethylation (129) also regulate PFKFB3 in cancer cells, decreasing its catalytic activity and redirecting the glycolytic flux to the PPP, increasing NADPH and decreasing ROS levels. Similarly, cell damage-mediated induction of p53 stimulates nucleotide biosynthesis by inhibiting *PFKFB3* expression and enhancing the flux of glucose through the PPP to increase nucleotide production, which promotes DNA repair and cell survival (130) (Figure 4).

The PFKFB3 isoenzyme can also be regulated through the ubiquitin-proteasome system (131), as it contains a KEN box that can be ubiquitinated by the E3 ubiquitin ligase of the anaphase-promoting complex (APC/C), which is activated by Cdh1, in a similar way to that of other proteins in the cell cycle. The proliferative response depends on the reduced activity of APC/C-Cdh1 to activate proliferation and glycolysis (132). PFKFB3 silencing prevents cell cycle progression, illustrating that this isoenzyme is essential for cell division (133). The tumor suppressor PTEN promotes APC/C-Cdh1 activity (127, 134) and cells from mice that overexpress PTEN show APC/C-Cdh1-mediated degradation of PFKFB3 and glutaminase, resulting in a decrease of glycolysis and proliferation, and an increase in resistance to oncogenic transformation (127). PFKFB3 isoenzyme levels have also been shown to increase in proliferating cells through the activation of cyclin D and E2F1, two downstream effectors of the PI3K-Akt-mTOR pathway (126), localizing in the nucleus and regulating proliferation through cyclin-dependent kinases (135). The nuclear targeting of PFKFB3 has been shown to increase cyclin-dependent kinase 1 (CDK1) expression among other cell cycle proteins. In particular, Fru-2,6-P_2_ stimulates the phosphorylation of the Cip/Kip protein p27 mediated by CDK1, which in turn elicits p27 ubiquitination and proteasomal degradation (136). PFKFB3 also interacts with CDK4, inhibiting its degradation via the ubiquitin proteasome pathway to promote cell cycle progression (137).

*PFKFB3* expression is induced by endotoxin in human macrophages (106, 124). Macrophage Toll-4 receptor agonists cooperate with adenosine to increase glycolysis by heightening *PFKFB3* gene expression and Fru-2,6-P_2_ synthesis, which increases glycolysis and favors ATP synthesis, developing the long-term defensive and reparative functions of macrophages (118). Macrophage glycolysis and pro-inflammatory activation mainly depend on HIF-1α and its effects on glucose uptake and the expression of hexokinase-II (HK-2) and *PFKFB3* (138) (Figure 4). These findings indicate that hypoxia enhances glycolytic flux in macrophages through HIF-1α and PFKFB3 proportionally to the upregulation of pro-inflammatory activities in these cells (138). These *in vivo* observations suggest that HIF-1α antagonists or PFKFB3 inhibitors could be used to alleviate inflammatory diseases. Furthermore, cytosolic viral recognition by secondary interferon signaling has been demonstrated to upregulate glycolysis preferentially in macrophages through PFKFB3 induction, promoting the extrinsic antiviral capacity of macrophages and being a crucial component of innate antiviral immunity (139).

*PFKFB3* is constitutively overexpressed in different cancer cell lines and in several human leukemias and solid tumors (140, 141), including ovarian and thyroid carcinomas (142), colon adenocarcinoma, breast cancer, gastric tumors and pancreatic cancer (73, 87, 111, 113, 143), and has been associated with lymph node metastasis and the TNM stage (143). PFKFB3 can also represent a biomarker and an anti-neoplastic target in gastric cancer (144). Furthermore, *PFKFB3* expression is required for cell growth and increased metabolic activity in myeloproliferative neoplasms expressing the oncogenic JAK2V617F kinase, which is a very common mutation in these malignancies. JAK2V617F and active STAT5 overexpress *PFKFB3* and *PFKFB3* silencing reduces cell growth under normoxic and hypoxic conditions and prevents tumor formation (145). In chronic myeloid leukemia (CML), PFKFB3 has been found to be strongly associated with resistance to the BCR-ABL tyrosine kinase inhibitors. *PFKFB3* silencing or pharmacological inhibition of its kinase activity enhances the sensitivity of CML cells to these inhibitors (146). In acute myeloid leukemia (AML), mTOR-mediated up-regulation of PFKFB3 is essential for cell survival, as mTORC1 up-regulates PFKFB3 in a HIF1α-dependent manner, and PFKFB3 silencing suppresses glycolysis and cell proliferation and activates apoptosis (147).

In malignant human colon tumors, PFKFB3 overexpression and phosphorylation of its S461 residue (P-PFKFB3 S461) has been observed (148). The cytokine IL-6 increases glycolysis by inducing *PFKFB3* expression through STAT3 activation (116), suggesting a functional role of PFKFB3 in chronic inflammation and in the development of colorectal cancer (117). Similar results have been reported in TGF-β1-induced lung fibrosis, in which PFKFB3 inhibition attenuates prefibrotic phenotypes and blocks the differentiation of lung fibroblasts (149). Furthermore, we have shown that TGF-β1 overexpresses *PFKFB3* mRNA and protein in glioblastoma cells through the activation of the Smad, p38 MAPK and PI3K/Akt signaling pathways. Inhibiting PFKFB3 expression or activity significantly suppressed the ability of T98G cells to form colonies, which is one of the hallmarks of cell transformation (121).

High-grade astrocytomas also contain increased PFKFB3 protein levels (150). The expression of the PFKFB splice variant UBI2K4 prevents tumor cell growth, acting as a tumor suppressor in astrocytic tumors (151). Besides, loss of heterozygosity in 10p14-p15, which leads to the allelic deletion of UBI2K4, has been detected in 55% of glioblastomas and is associated with poor prognosis (152).

PFKFB3 has also a key role in the interaction between cancer cells and other cells in the TME. Endothelial cells (ECs) depend on glycolysis more than on oxidative phosphorylation for ATP synthesis and loss of PFKFB3 in ECs impairs vessel formation (52). Inhibition of glycolysis by silencing PFKFB3 expression or pharmacologically blocking its kinase activity has been observed to inhibit pathological angiogenesis, such as ocular and inflammatory disease, without causing systemic effects (153). Recently, an article reported that targeting PFKFB3 in ECs significantly impeded metastasis by normalizing tumor vessels and improved the delivery and efficacy of chemotherapy (154). Nuclear factor (erythroid-derived 2)-like 2 (Nrf2) regulates endothelial glycolysis and proliferation through the transcriptional regulation of PFKFB3, *VEGFA, FOXO1*, and *MYC* (155), with a positive correlation occurring between Nrf2, HIF-1α, and *PFKFB3* expression in breast cancer cells, and cancer patients with high *PFKFB3* expression showing poorer overall survival (156).

*PFKFB3* expression is also linked to hepatocellular carcinoma (HCC) growth. *PFKFB3* overexpression has been associated with a large tumor size and poor survival in patients, while *PFKFB3* knockdown inhibits HCC growth by reducing glucose consumption and impeding DNA repair, which leads to cell cycle arrest at the G2/M phase and apoptosis. Silencing PFKFB3 expression decreases Akt phosphorylation and reduces the expression of ERCC1, a protein involved in DNA repair (157). The combination of aspirin and sorafenib has been shown to perform a synergistic effect against liver cancer. *PFKFB3* overexpression, associated with high glycolytic flux, is frequently observed with sorafenib resistance, which can be overcome by aspirin. By inhibiting PFKFB3, sorafenib plus aspirin induce apoptosis in tumors without eliciting weight loss, hepatotoxicity and inflammation, suggesting that their combination may be an effective treatment for HCC (158).

A large number of studies have reported that increased *PFKFB3* expression promotes proliferation and carcinogenesis, indicating that its inhibition could be crucial for treating inflammation and cancer. Indeed, siRNA suppression of PFKFB3 has been reported to reduce cancer cell viability (159, 160) while small molecule inhibitors of the PFKFB3 isoenzyme have been developed (161).

#### PFKFB4

*PFKFB4*, firstly cloned from rat (162) and human testis (163), is a 44,332 bp gene composed of 14 exons. Several splice variants have been reported, with the PFK-2 core domain being conserved among all of them (162, 164). The *PFKFB4* gene encodes an isoenzyme that is expressed in the testis under the regulation of testosterone (165, 166). Moreover, it has been demonstrated that PFKFB4 mRNA and protein levels are regulated by hypoxia and glucose levels in different cancer cell lines from the prostate, liver, colon, bladder, stomach and pancreas (86, 87, 167–171). PFKFB4 is a prognostic marker in invasive bladder cancer (172), where its expression is activated by HIF-1α (171) (Figure 4). In hepatic cancer cell lines, sulforaphane-induced apoptosis was shown to decrease PFKFB4 protein expression and glucose consumption whereas HIF-1α induced *PFKFB4* expression under hypoxic conditions (173). *PFKFB4* expression is also controlled by heme-oxygenase-2 in HepG2 cancer cells (174). In HCC, upregulated Peroxisome proliferator-activating receptor γ (PPARγ) induces *PFKFB4* expression through the transcriptional activity of its promoter, regulating glycolysis and cell proliferation (175). High PFKFB4 mRNA and protein expression have been described in three different glioblastoma stem-like cell lines, with shRNA-mediated knockdown of *PFKFB4* promoting apoptosis (176) and no phenotypic effect occurring in PFKFB4-silenced normal neural stem cells. Furthermore, HIF1α-induced PFKFB4 mRNA expression correlates with glioma tumor grade (176).

PFKFB4 is required to balance glycolytic activity and antioxidant production to maintain the cellular redox balance in prostate cancer cells (169). PFKFB4 mRNA expression has been found to be greater in metastatic prostate cancer cells than in primary tumors. PFKFB4 silencing selectively increases Fru-2,6-P_2_ concentration in prostate cancer cells, suggesting that it mainly functions as a fructose-2,6-bisphosphatase in these particular cells. The increase in Fru-2,6-P_2_ levels should direct glucose 6-phosphate toward the glycolytic pathway, thereby reducing the activity of the PPP. This would explain why prostate cancer cells show lower NADPH and glutathione levels after PFKFB4 silencing, which results in enhanced oxidative stress and cell death (169). Furthermore, p53 decreases *PFKFB4* gene expression by binding to its promoter to mediate transcriptional repression via histone deacetylases. PFKFB4 depletion also attenuates biosynthetic activity and induced ROS accumulation and cell death in the absence of p53 (177).

In a study investigating the differences in glucose metabolism between two forms of prostate cancer, small cell neuroendocrine carcinoma (SCNC) was found to be more glycolytic than adenocarcinoma, CD44 being a key regulator of glucose metabolism. *PFKFB4* expression in benign prostate tissue was lower than that in the adenocarcinoma, and significantly higher in SCNC. CD44 ablation in SCNC cells reduced both mRNA and protein levels of PFKFB4 (178). Thus, CD44 can modulate the aggressive phenotype of prostate cancer cells by increasing *PFKFB4* expression (179).

PFKFB4 can also regulate autophagy by influencing the redox balance. In PC3 prostate cancer cells, PFKFB4 inhibition was observed to cause p62 accumulation, which is usually associated with the inhibition of autophagy. However, the autophagic flux was increased in these cells. The combination of antioxidants and PFKFB4 inhibition prevented p62 accumulation, which was instead mediated by Nrf2, thus avoiding autophagy. Hence, *PFKFB4* expression is required for appropriate ROS detoxification in these cells (180). It was recently found that epithelial and endothelial tyrosine kinase interacts with PFKFB4 modulating chemoresistance of small-cell lung cancer by regulating autophagy (181).

Solid malignant tumors of the breast present a higher *PFKFB4* expression compared to non-malignant tissue. In several breast cancer cell lines, *PFKFB4* expression increased upon exposure to hypoxia (87). PFKFB4 was recently shown to act as a protein kinase phosphorylating the oncogenic steroid receptor coactivator-3 (SRC-3) and enhancing its transcriptional activity to drive breast cancer (182). PFKFB4 suppression or ectopic expression of a phosphorylation-deficient S857A mutant of SRC-3 abolished SRC-3-mediated transcription. Mechanistically, SRC-3 phosphorylation increases its binding with the ATF4 transcription factor by stabilizing the recruitment of SRC-3 and ATF4 to target gene promoters. Functionally, PFKFB4-induced SRC-3 activation directs the glucose flux toward the PPP and the synthesis of purines by transcriptionally upregulating the expression of transketolase. *PFKFB4* or SRC-3 silencing inhibits breast tumor growth and metastasis (182).

Apart from the importance of PFKFB4 in regulating cancer cell glycolysis, its expression also determines the metabolic adaptation of non-tumor cells. In mitogen-stimulated rat thymocytes, ConA was shown to induce the expression of *PFKFB3* and *PFKFB4* as well as increase glycolysis, cell proliferation and protein synthesis. This supports a role for these two proteins in coupling glycolysis to cell proliferation in lymphoid tissues (119).

### TIGAR

The *c12orf5* gene was discovered during a computer-based analysis of microarray data trying to find novel p53-regulated genes that are activated in response to ionizing radiation (183). This gene was cloned and characterized, and named as TP53-Induced Glycolysis and Apoptosis Regulator (*TIGAR*) (184) (Figure 4). *TIGAR* is a target of p53 that becomes rapidly activated by low levels of stress. The human *TIGAR* gene consists of six exons spanning about 38,835 bp, coding for a unique mRNA transcript variant of 8.2 kb with a 813 bp coding sequence. *TIGAR* promoter contains two p53 binding sites, one upstream of the first exon and the other within the first intron, the latter being the most efficient (184). *TIGAR* can be induced by Nutlin-3, an antagonist of Mdm2 that increases p53 levels (185), radiotherapy (183, 186), glutamine (29), chemotherapy (187), UV light (187), TNFα, and radiotherapy mimetics (188) or by the Akt signaling pathway in response to the metabolic stress caused by *PFKFB3* knockdown (189). *TIGAR* expression can also be regulated in a p53 independent manner (186, 189) by linking the CRE-binding protein (CREB) to the *TIGAR* promoter (190). Another transcription factor, the specificity protein 1 (SP1), can bind to *TIGAR* promoter and is considered important for its basal activity (191). *TIGAR* can be induced in response to hypoxia in myocytes (192) and some studies have identified HIF-1α as a regulator of cytochrome C-oxidase-2 (*SCO2*) and *TIGAR* gene expression in response to hypoxia (193). SCO2, a metallochaperone that is involved in the biogenesis of cytochrome C oxidase subunit II, participates in the mitochondrial chain, it is also induced by p53 and its blockage leads to the glycolytic phenotype (194).

The human TIGAR protein is composed of 270 amino acids and has a molecular weight of 30 kDa. It contains a bisphosphatase active center in which two histidine residues, H11 and H198, and one glutamic acid, E102, are essential for its activity (184). TIGAR contains a catalytic domain similar to the histidine phosphatase superfamily of proteins with a histidine forming a transient phosphoenzyme during catalysis (195). This domain shares similarity with those of the phosphoglycerate mutase (PGAM) family of enzymes and with the bisphosphatase domain of PFK-2/FBPase-2 isoenzymes (196). TIGAR bisphosphatase activity hydrolyzes Fru-2,6-P_2_ into Fru-6-P, which can then enter in the PPP to synthesize NADPH and ribose-5-phosphate, thus reducing ROS and producing nucleotide precursors that are essential for biosynthesis, DNA repair and cell proliferation (184) (Figure 4). As TIGAR has no kinase domain, it behaves as a kinase-deficient PFKFB isoform. Thus, cells overexpressing FBPase-2 show similar enhanced PPP flux and resistance to oxidative stress (197). The FBPase catalytic activity of TIGAR is several orders of magnitude lower than that of the FBPase-2 component of PFK-2/FBPase-2 isoenzymes, pointing out that Fru-2,6-P_2_ could not be its main physiological substrate (198).

TIGAR mRNA is expressed in all the tissues in which it has been analyzed to date and is overexpressed in several cancer cells. It localizes mainly in the cytoplasm, but has been observed to relocalize to the outer mitochondrial membrane under hypoxic conditions to form complexes with HK-2, limiting ROS production (199).

TIGAR has also been linked to autophagy. For example, TIGAR overexpression and reduced ROS levels have been observed alongside suppressed autophagy in cells exposed to stress conditions, and TIGAR suppression induced autophagy that subsequently mediates apoptosis by restraining ROS levels (200). The relationship between autophagy and apoptosis is regulated distinctively according to the stimulus and cell type. Thus, treatment of neuroblastoma cells with D-galactose induces necroptosis and autophagy, as reflected in the upregulation of *BMF, BNIP3, ATG5*, and *TIGAR*, without affecting the expression of the genes associated with apoptosis (201). Decreased mRNA levels of TIGAR and reduced levels of the damage-regulated autophagy modulator (DRAM) have been reported in HepG2 cells exposed to high oxidative stress or nutrient starvation (202). Upon disruption of the homeostasis balance of the cell, TIGAR is activated and provides protection through its antioxidant properties rather than by inhibiting autophagy, while other transcriptionally activated targets, such as DRAM, enhance autophagy (203). Some studies have proposed that p53 regulates stress-induced autophagy by balancing TIGAR and DRAM, which have opposite effects (204, 205).

Like the PFKFB isoenzymes, TIGAR can also play a role in cancer. The function of TIGAR in a specific cell type depends on the metabolic state of the cell and PFKFBs activities that determine Fru-2,6-P_2_ concentration and glycolytic flux. TIGAR activity could limit glycolysis and produce antioxidant molecules and precursors for nucleotide synthesis, thereby limiting cancer development. However, *TIGAR* overexpression can also promote the growth of tumor cells with high ROS levels. TIGAR has been reported not be necessary for normal growth and development in mice, but plays an important function in intestinal regeneration. The lack of TIGAR causes growth defects which are recovered by ROS scavengers and nucleosides (206). Besides, *TIGAR* deficiency has been reported to reduce tumor growth and improve survival in a mouse intestinal adenoma model, while elevated *TIGAR* expression supported cancer progression (206). *TIGAR* expression is increased in human breast, gastric and lung cancer, inversely correlating with p53 expression levels (207–209). Furthermore, TIGAR downregulation inhibits growth in several cancer cell lines (184). In a model of nasopharyngeal cancer, 1-(3-C-ethynyl-beta-d-ribo-pentofuranosyl)cytosine (ECyd), an RNA-nucleoside anti-metabolite with potent anticancer activity, was shown to downregulate TIGAR and deplete NADPH. *TIGAR* overexpression was able to recover the growth inhibition induced by ECyd (210). In the same model, c-Met protein kinase maintained *TIGAR* expression, whereas c-Met silencing significantly decreased *TIGAR* expression and subsequently depleted intracellular NADPH, which lead to cell death (211). TIGAR silencing induces also apoptosis and autophagy in HepG2 cells (212), while RNAi-mediated knockdown of citrate synthase in human cervical carcinoma cells accelerates cancer cell metastasis and proliferation deregulating the p53/TIGAR pathway (213). In HeLa cervical carcinoma cells, TIGAR can be induced in an Akt-dependent manner in response to the inhibition of glycolysis. PFKFB3 depletion by RNAi increases ROS levels and decreases cell viability, this effect being highly exacerbated when TIGAR is also inhibited. However, TIGAR inhibition alone does not have an impact on HeLa cell survival (189). Furthermore, some studies have reported that TIGAR regulates the cell cycle by de-phosphorylating the retinoblastoma protein (RB) and stabilizing RB-E2F1 complex, thus delaying entry into the S phase (187, 214).

In multiple myeloma cells, inhibition of MUC1-C oncoprotein increases ROS levels and downregulates *TIGAR* expression, resulting in decreased NADPH and glutathione levels and promoting ROS-mediated apoptosis/necrosis (215). Sensitivity to fludarabine and p53-mediated *TIGAR* induction has been described in chronic lymphocytic leukemia. The sensitivity to fludarabine varied despite all patients presented wild-type p53 (216). Glioblastoma cells overexpress *TIGAR* which reduces cell death induced by restricting glucose and oxygen (217). These results indicate the potential therapeutic use of TIGAR as an antitumoral target (186). Similarly, *TIGAR* expression was found decreased with a sonodynamic therapy tested in a neuroblastoma cell model which decreases cell proliferation, possibly through increased ROS levels (218). In glioblastoma-derived cell lines, *TIGAR* abrogation increased radiation-induced cell destruction, providing a new therapeutic strategy that could be used to increase cell death in glial tumors, thus allowing the use of lower doses of radiotherapy. Gliomas are resistant to radiotherapy and to TNFα-induced killing. Radiation-induced TNFα increases radioresistance through nuclear factor κB (NFκB). Thus, the existence of an ATM-NFκB axis regulating TIGAR indicates its involvement in the inflammation and resistance to radiomimetics (188). It was recently reported that TIGAR regulates NF-κB activation by suppressing phosphorylation and activation of the upstream IKKβ, which occurs through a direct binding competition between NEMO and TIGAR for the linear ubiquitination assembly complex (LUBAC), preventing the linear ubiquitination of NEMO required for the activation of IKKβ and other downstream targets. Furthermore, a TIGAR mutant with impaired phosphatase activity was equally effective as wild-type TIGAR in inhibiting the linear ubiquitination of NEMO, IKKβ phosphorylation/activation and NF-κB signaling, indicating that the effect of TIGAR on NF-κB signaling is due to a non-enzymatic molecular function, that directly inhibits the E3 ligase activity of LUBAC (219).

**Figure 5:**
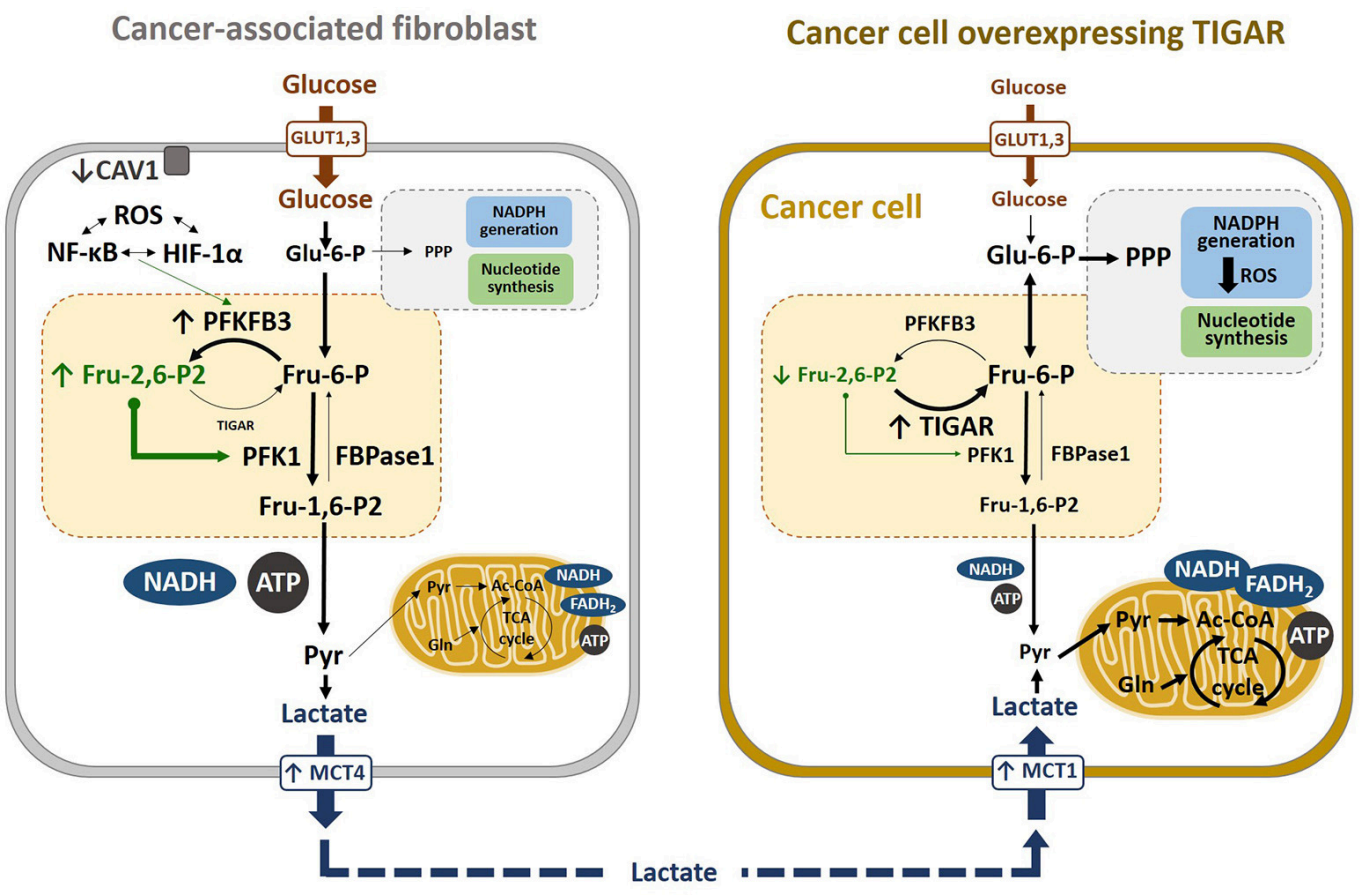
Metabolic reprogramming in cancer cells. Lactate transport is crucial in the crossed-regulation of metabolism between cancer-associated fibroblasts (CAFs) and cancer cells. In the fibroblasts close to carcinoma cells, ROS, HIF-1α, and NF-κB induce glycolysis, downregulating CAV1 and upregulating MCT4, which results in increased lactate secretion in CAFs. Conversely, TIGAR overexpression in carcinoma cells alters their metabolic state, increasing the pentose phosphate pathway (PPP). Lactate released from CAFs is taken by cancer cells via MCT1 and converted to pyruvate, which can enter the TCA cycle. These cancer cells have high mitochondrial OXPHOS and low glycolysis, which is associated with high proliferation and low apoptosis rates, resulting in increased tumor growth.

In a co-culture system, oxidative stress-induced autophagy correlated with caveolin-1 (CAV1) downregulation in CAFs and *TIGAR* overexpression in adjacent breast cancer cells (Figure 5). Reduced CAV1 expression in fibroblasts reduces mitochondrial function and induces glycolysis through HIF-1α and NF-kB signaling (30). Consequently, autophagic CAFs supply recycled substrates for the cancer cell metabolism and, also, avoid cancer cell death by overexpressing TIGAR, thereby conferring resistance to apoptosis and autophagy (220). Other studies by the same group have shown that the metabolic coupling between cancer cells and fibroblasts contribute to tamoxifen resistance, as CAFs enhanced TIGAR activity in cancer cells, that protected against tamoxifen-induced apoptosis (221). In another study, glutamine was described to increase TIGAR and be needed for CAV1 downregulation in CAFs, decreasing mediators and markers of autophagy in cancer cells. In this model, glutamine from autophagic fibroblasts may serve to fuel cancer cell mitochondrial activity. Thus, a cycle of nutrients between catabolic stromal cells and anabolic tumor cells has been suggested to account for the relationship between cells in the TME (19–21, 28–30, 222). In addition, *TIGAR* overexpression has been shown to reprogram carcinoma and stromal cells in breast cancer (29), as well as to increase oxygen consumption rates and ATP levels in the presence of glutamine and lactate, leading to enhanced ATP synthesis. Moreover, when carcinoma cells overexpress TIGAR in co-cultures with fibroblasts, a glycolytic phenotype is induced in the fibroblasts, inducing HIF-1α expression as well as increasing glucose uptake and the expression of *PFKFB3* and lactate dehydrogenase-A. *TIGAR* overexpression in carcinoma cells increases tumor growth and proliferation rates *in vivo* (29, 30). All these data support a two-compartment model of tumor metabolism (Figure 5). The mechanisms by which *TIGAR* overexpression increases mitochondrial activity remain unknown to date (29, 217).

### Fru-2,6-P_2_ metabolism and the regulation of glycolysis and the pentose phosphate pathway in cancer cells

The different PFKFB isoenzymes and TIGAR have important roles in the TME. The genes encoding these isoenzymes are commonly overexpressed in tumors and can be induced in response to cellular stress. These isoenzymes, regulating Fru-2,6-P_2_ concentration, have contrary effects on cell metabolism and different studies have dealt with the cross-regulation of these enzymes (Figure 4). The contribution of TIGAR to the regulation of Fru-2,6-P_2_ levels is expected to vary depending on the expression levels of the PFKFB1–PFKFB4 isoenzymes. *TIGAR* overexpression is associated with decreased survival of patients with acute myeloid leukemia (AML) (223) and other tumors (217, 224). Overexpression of *PFKFB2-4* has similar outcomes (97, 98, 113, 161, 176). However, crosstalk between these genes must exist in cancer cells since it has been observed that when *TIGAR* is eliminated in leukemia cells expressing high levels of *TIGAR, PFKFB3* expression increase, and when *TIGAR* is overexpressed in cells with low levels of *TIGAR, PFKFB3* expression decrease (223). Moreover, we have reported that *PFKFB3* silencing in HeLa cells elevates ROS levels and overexpresses *TIGAR* through Akt signaling, preserving against DNA damage and apoptosis (189). These results indicate that cancer cells sustain high glycolytic flux in order to fuel biosynthetic pathways under basal conditions. Nevertheless, if glycolysis is impeded, for example by ROS-induced S-glutathionylation or demethylation of PFKFB3 (128, 129), *PFKFB3* interference, or PFKFB3 inhibitors (189), cancer cells divert the flow toward the PPP. Cancer cells can also inhibit glycolysis to decrease ROS in response to DNA damage. In this respect, following DNA damage, the function of p53 reducing the mRNA and protein levels of PFKFB3 (130) and PFKFB4 (177) as well as the glycolytic flux, while increasing TIGAR expression and the levels of NADPH and nucleotides through the PPP (184, 186), enhances DNA repair and cell survival. In this sense, in p53-deficient cells exposed to UV radiation, glycolysis is not impeded supporting that p53 is required for this regulatory role (130, 177).

The FBPase catalytic efficiency of TIGAR has been reported to be several orders of magnitude lower than that of the FBPase-2 activity of PFKFBs (195, 198), thus challenging the concept that TIGAR acts primarily on Fru-2,6-P_2_. TIGAR could also exert its effects by directly increasing flux through the terminal part of glycolysis, given that it was found to act as the phosphoglycolate-independent 2,3-bisphosphoglycerate phosphatase (225), with 2,3-bisphosphoglycerate (2,3-BPG), 2-phosphoglycerate (2-PG) and phosphoenolpyruvate (PEP) being better substrates than Fru-2,6-P_2_ (198). These effects could also be enhanced by the presence of PKM2 in cancer cells, which has low affinity for PEP and provides a large amount of metabolic precursors for biosynthesis (14, 226). The concentration of 2,3-BPG in cells is three orders of magnitude lower than that in erythrocytes (227) and very little is known about its function, apart from the fact that it is an essential cofactor for the phosphoglycerate mutase. This adds a novel layer of complexity to the function of TIGAR that should be taken into account in future studies.

*PFKFB4* expression is essential for prostate and p53-null colon cancer cell survival, maintaining the balance between the use of glucose for energy generation and the synthesis of antioxidants (177). PFKFB4 silencing increases Fru-2,6-P_2_ levels in prostate cancer cells, suggesting that it mainly functions as a fructose-2,6-bisphosphatase in these particular cells, diverting glucose 6-phosphate toward the PPP (169). By contrast, *PFKFB4* that is expressed in other types of transformed cells and tumors synthesizes Fru-2,6-P_2_ and is required for the glycolytic reprogramming of cancer cells (170). Specific analyses of the enzymatic activity and regulation of PFKFB4 are needed to characterize its potential role as a FBPase-2 proposed in some studies (169, 177).

PFKFB4 has been shown to act as a protein kinase of SRC-3 resulting in the upregulation of transketolase (182). This finding could explain the effect of *PFKFB4* overexpression in some cancer cells, such as the transcription of a key non-oxidative enzyme of the PPP and the redirection of glycolytic intermediates to the non-oxidative arm of PPP.

Other inhibitory glycolytic effects that contribute to cancer development, such as the glycosylation of PFK1 in response to hypoxia (59) and the Snail repression of PFK-P (60), redirect glucose flux through the PPP, thereby conferring a selective growth advantage to cancer cells. Furthermore, FBPase1 overexpression suppresses cancer cell growth (65), its loss correlating with advanced tumor stage and poor prognosis (66). Snail can also repress FBPase1 in breast cancer cells (67), regulating glucose flux toward glycolysis or the PPP by suppressing either FBPase1 or PFK-P, respectively.

Finally, FBPase1 and PFK-L are part of the “glycosome,” a complex that modulates the activities of these enzymes and integrates them with others such as PKM2 and PEPCK1. Quantitative high-content imaging assays indicate that the direction of glucose flux between glycolysis, the PPP and serine biosynthesis seems to be spatially regulated by these multienzyme complexes in a cluster size-dependent manner, providing new mechanistic insight into how a cell regulates the direction of glucose flux between energy metabolism and anabolic biosynthesis (53).

## Targeting the tumor metabolic ecosystem

The ability to selectively modulate the metabolism of cancer cells could have high therapeutic potential. Tumor cells expressing active oncogenes and/or defects in their tumor suppressors enter apoptosis when glucose oxidation is limited. Peculiarly, these same cells often show resistance to other forms of apoptotic stimuli (radiation and chemotherapy) and the use of glycolytic inhibitors sensitizes cells to these stimuli and promotes their death (21, 228, 229).

The dependence of cancer cells on glucose consumption led to the development of different therapeutic approaches. Pharmacological inhibition of glycolysis has emerged as a novel strategy since high glycolytic activity is considered a metabolic hallmark of cancer (6, 21, 31, 34). HIF-1α overexpression and the induction of glycolytic isoenzymes, present in many tumors, have been shown to generate resistance to chemotherapy and radiation (39, 230, 231). Therefore, inhibiting HIF-1α could be an important component of cancer therapy (232, 233).

One of the most studied inhibitors has been 2-DG, a glucose molecule in which the 2-hydroxyl group has been replaced by hydrogen. 2-DG can be phosphorylated by hexokinase, but it cannot be metabolized by phosphohexose-isomerase. Therefore, its intracellular accumulation produces a competitive inhibition of hexokinases. 2-DG has cytotoxic effects on different types of cancer cells, especially those overexpressing HIF-1α and with mitochondrial defects (229, 234). Accordingly, 2-DG significantly increases the response to treatment with adriamycin and paclitaxel in human osteosarcoma-bearing mice and of small cell lung cancer (234). However, the high doses needed to compete with glucose can induce toxicity (21, 235). Inhibition of other glycolytic enzymes has been shown to successfully suppress tumor cell growth, although systemic toxicity and lack of therapeutic benefit has precluded further development in numerous preclinical studies (21).

The fact that the *PFKFB2-4* genes are overexpressed in different tumors and are activated by hypoxia and/or oncogenes indicates that their role is necessary in the development of the glycolytic phenotype, facilitating the adaptation and survival of tumor cells in hypoxic micro-environments. Thus, small molecule inhibitors of PFKFBs could be used to improve the efficiency and specificity of cancer treatment (161, 236).

The expression of more than one PFKFB isoenzyme in some cells suggests the use of less specific PFKFB kinase inhibitors to effectively reduce Fru-2,6-P_2_ concentrations. In this sense, targeting of both PFKFB3 and PFKFB4 isoenzymes has been proposed to be advantageous due to their high expression in some cancer cells (173). PFKFB isoenzyme inhibitors could also be used in combination with agents that mimic hypoxic conditions, increasing cellular dependence on the upregulation of glycolysis and PFKFBs. The use of chemotherapeutic drugs together with PFKFB3 inhibitors may improve response rates as well as progression-free survival in cancer patients. This is corroborated by recent data demonstrating that sorafenib resistance in HCC can be overcome by aspirin, through PFKFB3 inhibition (158). This type of anti-glycolytic approach substantially differs from previous cancer treatments that attempted to block glycolysis entirely and in a permanent way, causing significant adverse effects. Given that Fru-2,6-P_2_ is not part of a main metabolic pathway, and is not a biosynthetic precursor or intermediate in energy production, its concentration can be independently controlled, making PFKFB isoenzymes more specific targets.

TIGAR has important functions in the regulation of cell processes such as apoptosis, autophagy, DNA repair, and the control of oxidative stress. The elevated levels of *TIGAR* expression in some types of tumors (207, 217) and the action of different therapeutic agents associated with decreased *TIGAR* expression, highlight the importance of *TIGAR* in tumor cell survival. TIGAR can support tumorigenesis by reducing ROS production and generating precursors for biosynthesis. These data indicate that the inhibition of TIGAR might confer advantages in cancer treatments (29, 186, 206, 221). Moreover, TIGAR silencing has been shown to increase sensitivity of glioblastoma cells to radiotherapy (186, 237) and increase cell death mediated by PFKFB3 inhibition (189).

The current results show that the metabolic phenotype of tumor cells is heterogeneous and that metabolic coupling occurs between different cell populations of the TME with complementary metabolic profiles. The metabolic differences between tumor and non-tumor cells can potentially be exploited therapeutically. There are currently no glycolytic inhibitors that have been approved as anticancer agents (21) and little is known about the degree of glycolysis inhibition in tumor vs. non-tumor tissues that can be achieved with glycolytic inhibitors, but the preclinical results obtained with these molecules look promising.

The targeting of mitochondrial oxidative metabolism and antioxidant effectors also hold promise as anticancer strategies. Arsenic trioxide, an inhibitor of the mitochondrial oxidative metabolism, has been approved for the treatment of acute promyelocytic leukemia (238). Metformin, an inhibitor of complex I of oxidative phosphorylation (239), has been shown to increase lactate levels and induce apoptosis in a clinical trial in head and neck squamous cell cancer (240). In the same clinical trial, metformin was shown to induce CAV1 expression in CAFs, preventing the metabolic coupling between stromal and cancer cells (240, 241). Another example of effective anti-metabolic cancer therapies is the use of N-acetyl cysteine (NAC), whose antioxidant potential reduced both the proliferation of cancer cells and the expression of the metabolic coupling monocarboxylate transporter 4 (MCT4) in stromal cells in a clinical trial in breast cancer (242). In summary, inhibiting oxidative metabolism or altering the redox state of tumors appear as promising approaches for the treatment of cancer.

## Conclusions

In this review, we summarize current knowledge on the enzymes regulating the Fru-6-P/Fru-1,6-P_2_ cycle and their role in cancer and TME cells. PFKFB and TIGAR enzymes control this cycle and are overexpressed in cancer cells, acting as prognostic markers. Small molecule inhibitors of PFKFB2-4 in combination with other drugs could increase the efficiency of cancer treatment. Further preclinical data on PFKFBs inhibitors are required to confirm their potential clinical use.

In summary, glycolysis in tumor cells is a complex phenomenon in which this and other metabolic pathways are reprogrammed to increase energy production and biomolecular synthesis required for cell proliferation. Understanding the regulation of genes and glycolytic isoenzymes in cancer cells and other cells of the TME will have implications for cancer diagnosis and prognosis and for the development of more selective therapies.

## Author contributions

All authors jointly developed the structure and arguments of the paper, prepared the manuscript, reviewed it and approved the final version. RB supervised each of the tasks.

### Conflict of interest statement

The authors declare that the research was conducted in the absence of any commercial or financial relationships that could be construed as a potential conflict of interest.

## Abbreviations

2,3-BPG: 2,3-bisphosphoglycerate
2-DG: 2-deoxy-D-glucose
AML: acute myeloid leukemia
AMPK: AMP-activated protein kinase
APC/C: anaphase-promoting complex
AR: androgen receptor
CAF: cancer-associated fibroblast
CAV1: caveolin-1
CDK1: cyclin-dependent kinase 1
CML: chronic myeloid leukemia
ConA: concanavalin A
CREB: CRE-binding protein
DRAM: damage-regulated autophagy modulator
EC: endothelial cell
ECyd: 1-(3-C-ethynyl-beta-d-ribo-pentofuranosyl)cytosine
ER: estrogen receptor
ERE: estrogen response element
FBPase1: fructose 1,6-bisphosphatase
FBPase-2: fructose 2,6-bisphosphatase
Fru-1, 6-P_2_: fructose-1,6-bisphosphate
Fru-2, 6-P_2_: fructose 2,6-bisphosphate
Fru-6-P: fructose-6-phosphate
GASC: glioblastoma-associated stromal cell
GC: glucocorticoid
Glut1: glucose transporter 1
HCC: hepatocellular carcinoma
HIF-1α: hypoxia inducible factor-1α
HK-2: hexokinase-II
IL-6: interleukin-6
LPS: lipopolysaccharide
LUBAC: linear ubiquitination assembly complex
MACC1: metastasis-associated in colon cancer protein 1
MAPK-1: mitogen-activated protein kinase 1
MCT4: metabolic coupling monocarboxylate transporter 4
NFκB: nuclear factor κB
PDK1: pyruvate dehydrogenase kinase
PEP: phosphoenolpyruvate
PEPCK1: phosphoenolpyruvate carboxykinase 1
PET: positron emission tomography
PFK1: 6-phosphofructo-1-kinase
PFK1/FBPase1: 6-phosphofructo-1-kinase/fructose 1,6-bisphosphatase
PFK-2: 6-phosphofructo-2-kinase
PFK-2/FBPase-2: 6-fosfofructo-2-kinase/fructose 2,6-bisphosphatase
PGAM: phosphoglycerate mutase
PKA: cAMP-dependent protein kinase
PKB: protein kinase B
PKM2: pyruvate kinase isoenzyme M2
PP-1: protein phosphatase 1
PPARγ: peroxisome proliferator-activating receptor γ
PPP: pentose phosphate pathway
PR: progesterone receptor
PRE: progesterone response element
RB: retinoblastoma
ROBO1: round about guidance receptor 1
ROS: reactive oxygen species
RSK: p90 ribosomal S6 kinase
SCNC: small cell neuroendocrine carcinoma
SCO2: cytochrome C-oxidase-2
SLIT2: slit guidance ligand 2
SP1: specificity protein 1
SRC-3: steroid receptor coactivator-3
TGF-β1: transforming growth factor beta 1
TIGAR: TP53-induced glycolysis and apoptosis regulator
TME: tumor microenvironment
VEGF: vascular endothelial growth factor.
